# Comparison of Anti-factor Xa Levels in Female and Male Patients with Obesity After Enoxaparin Application for Thromboprophylaxis

**DOI:** 10.1007/s11695-021-05875-z

**Published:** 2022-01-05

**Authors:** Jonas Wagner, Henrike Wruck, Anne Lautenbach, Philipp von Kroge, Stefan Wolter, Oliver Mann, Jakob Izbicki, Anna Duprée

**Affiliations:** 1 Department of General, Visceral and Thoracic Surgery, University Medical Center Hamburg-Eppendorf, Martinistr. 52, 20246 Hamburg, Germany; 2 III. Department of Medicine, University Medical Center Hamburg-Eppendorf, Martinistr. 52, 20246 Hamburg, Germany

**Keywords:** Bariatric surgery, Anti-factor Xa activity, Enoxaparin, Prophylaxis, Venous thromboembolism, Sex difference

## Abstract

**Purpose:**

Venous thromboembolic events (VTEs) are common complications after bariatric surgery, and enoxaparin is commonly used to prevent VTEs. The risk for VTEs is sex-specific. Whether enoxaparin application results in similar anti-factor Xa activities (aFXa) in males and females with obesity remains to be determined. We investigated whether our dosage regimen of enoxaparin resulted in similar serum aFXa levels in female and male patients undergoing bariatric surgery.

**Materials and Methods:**

We administered enoxaparin twice daily in patients undergoing bariatric surgery. Patients with a body mass index (BMI) > 60 kg/m^2^ (*n* = 11) received 60 mg enoxaparin (group 2), and patients with lower BMI (*n* = 86) received 40 mg per dose (group 1). Peak aFXa levels were measured 3 days after surgery. The primary outcome was the aFXa level. As a secondary outcome, we detected VTEs and major bleeding events and explored the possible influencing factors of aFXa.

**Results:**

Women had higher aFXa than men, but after matching for anthropometric values, the two groups were similar (females: 0.17 ± 0.08 U/ml; males: 0.18 ± 0.08 U/ml). Linear regression revealed a moderate relationship between weight and aFXa levels. The 3-month follow-up was attended by 94.9%, at which one patient had pulmonary embolism.

**Conclusion:**

Individual enoxaparin dosage regimens for men and women do not seem to be required. Weight-based dosing regimen seems to be a more reasonable choice.

**Graphical abstract:**

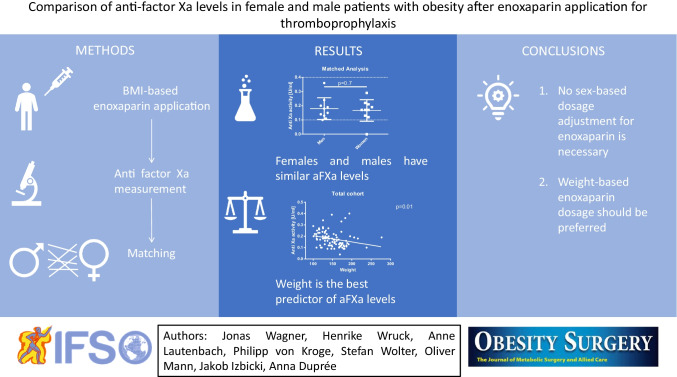

## Introduction

Venous thromboembolism (VTE) is a common complication following surgery and is accompanied by a high mortality rate [1]. The incidence of VTEs ranges from 0.8–3.2% after bariatric surgery [2]. Risk factors for VTE include surgery, old age, cancer, trauma, personal history of VTE, and obesity [3]. Many surgical patients have a moderate to high risk for developing VTE, so chemoprophylaxis is indicated. Enoxaparin, a low-molecular-weight heparin (LMWH), is one of the most common drugs used for thromboprophylaxis [4]. For most normal-weight surgical patients, a fixed dosage of 40 mg enoxaparin subcutaneously is sufficient for reliable thromboprophylaxis [5]. In patients with obesity, there is insufficient evidence on the optimal dosage for thromboprophylaxis with enoxaparin, though they have an increased risk for VTE [6]. VTEs are cardiovascular diseases (CVDs) [3], and the risk for CVDs, including VTEs, is sex-specific [7].

Sex has a major impact on many different pharmacological responses, yet it has not been recognized as important factor. Many different aspects could influence pharmacokinetics in a sex-specific way, such as differences in total water, plasma volume, metabolism, or muscle content between men and women [8]. Fat distribution throughout the human body also shows sexual dimorphism [9, 10]. Male and female tissues show sex-specific differences, including in adipose tissue, where some metabolic genes are more highly expressed in females [11]. Enoxaparin is injected subcutaneously, and all of these factors might be able to influence the anti-factor Xa (aFXa) activity level in a sex-specific way.

Therefore, we investigated whether the application of the same enoxaparin dose leads to similar anti-factor Xa levels in male and female patients with obesity. We also sought to determine the possible factors that influence the aFXa level.

## Materials and Methods

### Patient Selection

We conducted this prospective study of patients undergoing bariatric surgery in our Center of Excellence for bariatric surgery. We included all patients between April 2019 and May 2020 for whom anti-factor Xa activity was available. We excluded patients who were currently receiving therapeutic anticoagulation or had chronic kidney disease. Data regarding height, weight, BMI, waist circumference, comorbidities, type of surgery, and standard laboratory parameters were collected. We use a BMI-based enoxaparin dosage as our in-house standard, since the optimal dosage of enoxaparin for patients with obesity has yet to be determined [12–14]. Patients were administered enoxaparin (Clexane®/Sanofi-Aventis) subcutaneously twice daily as thromboprophylaxis based on their BMI. Enoxaparin administration started 6 h after surgery. Sixty milligrams enoxaparin per dose was administered to patients with BMI above 60 kg/m^2^ (group 2), while patients with lower BMI received 40 mg per dose (group 1). Anti-factor Xa activity was measured 4–5 h after enoxaparin application on the third day after surgery. An anti-factor Xa activity between 0.1 and 0.4 U/mL was chosen as the target range for thromboprophylaxis, according to the literature [15–19] and our local laboratory values. The anti-factor Xa assay quantitatively measures the amount of factor Xa left in the sample in a chromogenic assay and is inversely proportional to the amount of LMWH [6]. It is used to monitor the effect of LMWH in special situations, such as obesity [7]. Patients left on enoxaparin for 28 days. Perioperative data were available for all 97 patients. Certified bariatric surgeons performed all surgical procedures. All patients were screened before surgery by a multidisciplinary team consisting of an endocrinologist, psychologist, nutritionist, physical therapist, and surgeon. Patients were selected for surgery if they had BMI ≥ 40 kg/m^2^ or if they had BMI ≥ 35 kg/m^2^ and related comorbidities in accordance with the German Guidelines of Surgical Treatment of Obesity after discussion with our interdisciplinary obesity board. The operating surgeon decided which procedure to perform depending on the BMI, comorbidities, medication, and patient’s request [20].

The primary outcome was anti-factor Xa activity. As a secondary outcome, we detected VTEs and major bleeding events [21] and explored possible influencing factors of aFXa. Bleeding was assessed by clinical evaluation and laboratory tests. We matched male and female patients 1:1 based on weight, BMI, waist circumference, and age.

Our institutional review board approved this study. All patients gave their informed consent.

### Statistics

We performed statistical analysis with the Statistical Package for Social Sciences software (SPSS; IBM, Version 24) and GraphPad Prism (GraphPad Software, Inc., Version 6). Patient characteristics are presented overall as the mean ± SD for continuous variables. For comparisons between continuous variable groups, the independent Student’s *t*-test was performed. We used the χ^2^ test to analyze differences between nominal data. Linear regression analysis was conducted to detect the relationship between aFXa, BMI, weight, and waist circumference. *p*-values < 0.05 were considered to be statistically significant.

## Results

### Patient Characteristics, Anti-factor Xa activity, and Complications

The baseline characteristics of all included patients are displayed in Table 1. We analyzed 97 patients in this study. The mean weight was 150 ± 30.9 kg, and the mean BMI was 50.6 ± 8.5 kg/m^2^. Of the 97 patients, 63 were women (64.9%), and the most common procedure was sleeve gastrectomy (74.2%). One patient (1%) had formally elevated creatinine levels, but enoxaparin dosage adjustment was not needed. Five patients had a history of cancer (5.2%), two had previous VTEs (2.1%), and one was paralyzed and had reduced mobility (1%) (Table 1). Both groups had similar baseline characteristics apart from weight, BMI, and waist circumference (Table 2).

**Table 1:** Patient characteristics

*n*	97
Age [years]	42 ± 11
Weight [kg]	150 ± 30.9
Height [cm]	172 ± 10
BMI [kg/m^2^]	50.6 ± 8.5
Waist circumference [cm]	145 ± 15.5
Women [*n*/%]	63/64.9
Men [*n*/%]	34/35.1
Procedure:	
•Sleeve gastrectomy [n/%]	72/74.2
•Gastric bypass [n/%]	22/22.7
•Other_1_ [n/%]	3/3.1
Anti-factor Xa activity [U/ml]	0.18 ± 0.07
Diabetes mellitus [*n*/%]	53/54.6
Hypertension [*n*/%]	51/52.6
OSAS [*n*/%]	24/24.7
Elevated serum creatinine [*n*/%]	1/1
History of cancer [*n*/%]	5/5.2
Antiphospholipid syndrome [*n*/%]	0/0
Estrogen therapy [*n*/%]	0/0
History of VTE [*n*/%]	2/2.1
Autoimmune disease [*n*/%]	0/0
History of heparin induced thrombocytopenia [*n*/%]	0/0
Central venous catheter or pacemaker [*n*/%]	0/0
Paresis or paralysis [*n*/%]	1/1
Major bleeding event [*n/*%]	0/0

_1_Other procedures included conversion of sleeve-gastrectomy to gastric bypass and limb distalization

**Table 2:** Patient characteristics, comparison of the two groups

	Group 1 (BMI < 60 kg/m^2^)	Group 2 (BMI ≥ 60 kg/m^2^)	*p* value
*n*	86	11	
Age [years]	42.8 ± 11	36.8 ± 9	0.06
Weight [kg]	143.7 ± 24.5	197.6 ± 34.8	< 0.001
Height [cm]	172 ± 10.5	171 ± 9	0.9
BMI [kg/m^2^]	48.5 ± 5.7	67 ± 8.5	< 0.001
Waist circumference [cm]	142 ± 14	164 ± 13	< 0.001
Women [n/%]	56/65.1	7/63.6	1
Men [n/%]	30/34.9	4/36.4	1
Procedure:			0.1
• Sleeve gastrectomy [n/%]	62/72.1	10/90.9	
• Gastric bypass [*n*/%]	22/25.6	0/0	
• Other_1_ [*n*/%]	2/2.3	1/9.1	
Anti-factor Xa activity [U/ml]	0.18 ± 0.07	0.18 ± 0.08	0.71
Diabetes mellitus [*n*/%]	47/54.7	6/54.5	0.99
Hypertension [*n*/%]	46/53.5	5/45.5	0.62
OSAS [*n*/%]	22/25.6	2/18.2	0.8
Elevated serum creatinine [*n*/%]	1/1	0/0	0.72
History of cancer [*n*/%]	4/4.7	1/9.1	0.54
History of VTE [*n*/%]	2/2.4	0/0	0.61
Paresis or paralysis [*n*/%]	1/1.2	0/0	0.72

_1_Other procedures included conversion of sleeve-gastrectomy to gastric bypass and limb distalization

As the primary outcome, aFXa was 0.18 ± 0.07 U/ml. Ninety (93%) patients achieved the aFXa target value of at least 0.1 U/ml. We measured an aFXa level of 0.18 ± 0.08 U/ml in group 2, which was not significantly different from that in group 1 (0.18 ± 0.07 U/ml) (Table 2).

One patient (1%) developed pulmonary embolism, although this patient’s aFXa level was above 0.2 U/ml. None of our patients had a major bleeding event. The 3-month follow-up data were available for 92 patients (94.8%), and none showed signs of a new VTE.

### Male and Female Patients Have Similar aFXa Levels

**Fig. 1:**
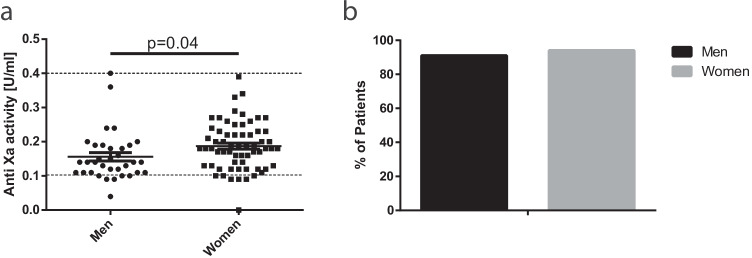
Mean anti-factor Xa activity ± SD [U/ml] in men and women (**a**). % of patients, who achieved target range (**b**)

**Fig. 2:**
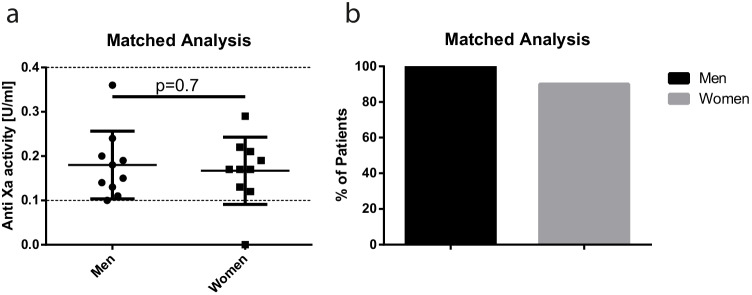
Mean anti-factor Xa activity ± SD [U/ml] in matched males and females (**a**). % of patients, who achieved target range (**b**)

We then addressed our main question of whether males and females might have different aFXa levels. In males, we measured an aFXa level of 0.16 ± 0.07 U/ml, while females had a slightly higher level of 0.19 ± 0.07 U/ml (Fig. 1a). Both groups achieved the target range at similar rates (Fig. 1b). Their aFXa difference was significant (*p* = 0.04); however, the female group was significantly lighter in weight (Table 3). We matched males and females based on age, weight, BMI, and waist circumference to reduce the potential bias. Matching resulted in 20 patients, who had no difference in anthropometric measures (Table 4). We observed almost identical aFXa levels in the matched pairs of patients (Fig. 2a). All of these male subjects achieved the target aFXa, while 90% of these females reached our goal (Fig. 2b).

**Table 3:** Characteristics of males and females

	Men	Women	*p* value
n	34	63	
Age [years]	40.5 ± 11	43.1 ± 11	0.27
Weight [kg]	170 ± 26	139 ± 28	< 0.001
Height [cm]	182 ± 7	166 ± 7	< 0.001
BMI [kg/m^2^]	51 ± 8	50 ± 9	0.5
Waist circumference [cm]	155 ± 12	139 ± 14	< 0.001
Anti-factor Xa activity [U/ml]	0.16 ± 0.07	0.19 ± 0.07	0.04
Diabetes mellitus [*n*/%]	23/67.6	30/47.6	0.06
Hypertension [*n*/%]	19/55.9	32/50.8	0.63
OSAS [*n*/%]	15/44.1	9/14.3	0.01
Elevated serum creatinine [*n*/%]	0/0	1/1.6	0.46
History of cancer [*n*/%]	1/2.9	4/6.3	0.46
History of VTE [*n*/%]	0/0	2/3.2	0.29
Paresis or paralysis [*n*/%]	1/2.9	0/0	0.17

**Table 4:** Characteristics of matched males and females

	Men	Women	*p* value
n	10	10	
Age [years]	45.5 ± 11	47.3 ± 10	0.71
Weight [kg]	147 ± 24	146 ± 25	0.93
Height [cm]	176 ± 5	170 ± 8	0.054
BMI [kg/m^2^]	47.2 ± 7	50.2 ± 7	0.37
Waist circumference [cm]	148 ± 15	143 ± 15	0.45
Anti-factor Xa activity [U/ml]	0.18 ± 0.08	0.17 ± 0.08	0.7
Diabetes mellitus [*n*/%]	8/80	5/50	0.16
Hypertension [*n*/%]	6/60	5/50	0.65
OSAS [*n*/%]	7/70%	2/20%	0.025
Elevated serum creatinine [*n*/%]	0/0	1/10	0.31
History of cancer [*n*/%]	1/10	0/0	0.31
History of VTE [*n*/%]	0/0	0/0	
Paresis or paralysis [*n*/%]	0/0	0/0

### Linear Regression Reveals that Weight is a Better Predictor of aFXa than BMI and Waist Circumference

**Fig. 3:**
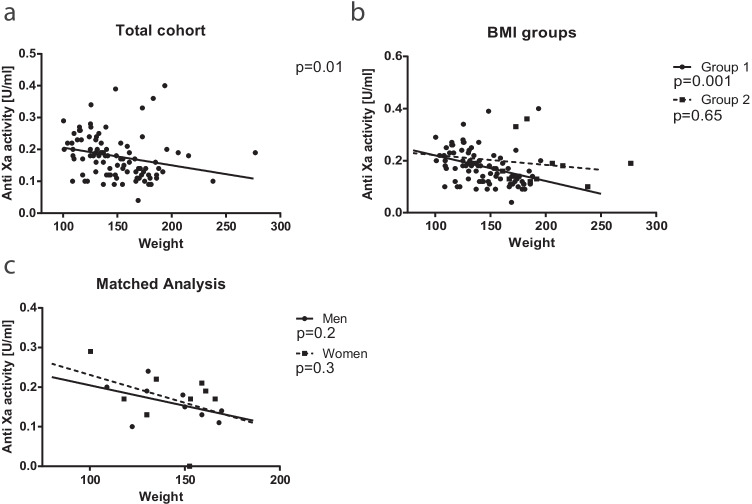
Scatter plot of weight [kg] and anti-factor Xa activity [U/ml] and linear regression for all patients (**a**), the two BMI-groups (**b**), and in the matched groups (**c**)

To determine parameters that might influence the aFXa levels, we performed simple linear regression analysis with weight, BMI, and waist circumference as independent variables. For the total cohort, only weight significantly impacted aFXa levels (*p* = 0.01), with a coefficient of determination (R^2^) of 0.06 (Fig. 3a). We performed linear regression using weight as an independent variable in groups 1 and 2 to assess the impact of BMI-based dosage. We observed a significant relationship between weight and aFXa only in group 1 (R^2^ = 0.12) (Fig. 3b). Next, we performed regression analysis only in our matched groups and excluded the matching pair of patients from group 2. According to the results, weight was not an independent predictive variable in the matched cohorts (Fig. 3c).

## Discussion

Recurring VTEs are more frequent in men [22]. Recently, evidence has emerged that men have a higher risk of VTE than women [7]. Considering the differences in total water, plasma volume, metabolism, muscle content, or adipose tissue distribution between men and women [8], it is natural to assume that men and women also have different aFXa levels after enoxaparin application. Therefore, the main research question we asked was whether male and female patients undergoing bariatric surgery have similar aFXa levels. Initially, we found that females had higher aFXa levels. Because females were significantly lighter than males, we matched males and females based on anthropometric measurements. Ultimately, after matching, we detected almost identical aFXa levels between men and women. We concluded that males and females with obesity indeed have similar anti-factor Xa activity levels after enoxaparin application.

Many previous studies have tested different dosages of enoxaparin for patients with obesity [12–14, 19, 23, 24]. Most of them focused on the optimal dosage regimen while not taking sex into account. Only Gelikas et al. reported that females had significantly higher aFXa levels than men. As in our study, the female population of Gelikas et al. was significantly lighter, which led them to conclude that this difference might be due to different weights [12], but they lacked proof of this claim. By matching the males and females of our study cohort, we indeed showed that there was no difference in aFXa levels between males and females with obesity. This is a very important finding, considering that the “best dosage” for patients with obesity has yet to be determined [12–14]. Future studies should now be able to determine the best dosing strategy without having to account for the different sexes.

Most of our subjects achieved our target range of 0.1–0.4 U/ml. Previous studies used different prophylactic ranges from ours, some of them starting at 0.2 U/ml [13] or even 0.5 U/ml [12]. The optimal prophylactic aFXa range is under debate [15]. Undisputedly, patients undergoing bariatric surgery need postoperative thromboprophylaxis and should be treated for another 28 days. Otherwise, their risk for VTEs is high even after discharge [25]. If patients do not reach the desired level, the consequences of this failure are not completely understood. Karcutskie et al. observed no difference in the incidence of VTE in trauma patients who reached their target compared to those who did not [26]. However, a better marker has yet to be established, and an aFXa level of 0.1 U/ml might be just as good as 0.2 U/ml [27].

Our second aim was to detect VTEs and major bleeding events. None of our patients showed signs of bleeding. One patient developed symptomatic PE and needed therapeutic anticoagulation. Rocha et al. reported that the postoperative pulmonary embolism rate was between 0.8 and 3.2% in bariatric surgical patients [2]. Although a rare event, PE and its consequences can be severe. The PE patient in our study had a history of DVT, but anticoagulation was no longer indicated. This patient had no other VTE risk factors and the aFXa level was above 0.2 U/ml. However, a DVT history, surgery, and obesity are all risk factors for developing VTE [28]. Therefore, bariatric patients with additional risk factors should be monitored more closely. Additionally, clinicians should focus preoperatively on acquiring a complete patient history to know who is at risk. This PE event further underlines the need for another marker for thromboprophylaxis.

Lastly, we also showed that weight was a better predictor for aFXa than BMI, which is in line with previous studies [12–14]. Enoxaparin’s volume of distribution is almost equal to the blood plasma [29] and weight might simply be the best predictor even for patients with obesity. For patients with BMI above 60 kg/m^2^, weight was not predictive of aFXa, and close surveillance seems to be indicated. We also wondered whether there might be a sex-specific effect present. We observed no significant relationship between aFXa and weight. However, by looking at both matched male and female graphs, it is evident that both have the same course as the overall cohort. Therefore, one can only conclude that this significant relationship should exist independently of sex. All graphs indicate that, with a weight above 150 kg, aFXa tends to be below 0.2 U/ml. Physicians should consider checking aFXa levels and signs of VTE regularly for patients with high weight (> 150 kg), BMI above 60 kg/m^2^, or additional risk factors.

We are aware that this study has some limitations. The aFXa level might not be the best marker for measuring the efficacy of thromboprophylaxis. A more clinical approach might be better suited, for example, a comparison of the aFXa level with sonography of the major leg veins or even computed tomography angiography (CTA) of the chest. These approaches are time-consuming and expensive but also might mean unnecessary radiation exposure for the patient. Therefore, we chose to measure the aFXa level. Furthermore, group 2 consisted of only 11 patients. We are aware that drawing conclusions from a rather small number of patients is difficult, but our main finding that a sex-specific dosage regimen is not required is based on all 97 patients. Hence, this conclusion can be deemed robust.

## Conclusion

Enoxaparin application leads to similar anti-factor Xa activity levels in male and female patients with obesity. We further conclude from our data that weight-based dosing of enoxaparin should be preferred in patients with obesity. Given the shortcomings of aFXa levels, additional close clinical monitoring might be needed for the surveillance of high-risk patients. Further studies are still needed to determine the optimal dosage regimen but do not have to take sex into account.
